# Spontaneous Retroperitoneal Hematoma in the Setting of Myasthenic Crisis

**DOI:** 10.7759/cureus.11116

**Published:** 2020-10-23

**Authors:** Joseph A Galgano, Michelle Bernshteyn, Pratibha Kaul

**Affiliations:** 1 Internal Medicine, State University of New York Upstate Medical University, Syracuse, USA; 2 Critical Care Medicine, State University of New York Upstate Medical University, Syracuse, USA

**Keywords:** retroperitoneal hematoma, iliopsoas hematoma, anticoagulation, major bleeding events, myasthenia gravis, myasthenic crisis

## Abstract

Our case highlights a patient with spontaneous retroperitoneal hematoma without clear cause in the setting of myasthenic crisis. While myasthenia gravis (MG) has been reported in the literature to be associated with vascular pathology such as polyarteritis nodosa, its association with coagulopathy and spontaneous major bleed is currently unclear. The patient in this case developed a sudden unprovoked iliopsoas hematoma while in the ICU for the management of newly diagnosed MG. Acute anemia was the only clinical sign which was later confirmed by imaging findings.

## Introduction

Major bleeding events are rare complications in the ICU setting but are associated with significant mortality. The incidence of spontaneous retroperitoneal hematoma is as low as 3.8 cases per 1000 ICU admissions, making it a relatively rare condition to encounter [1]. Most commonly, they are associated with trauma. Clinical signs and symptoms including abdominal or back pain, ecchymosis, and palpable mass are often difficult to appreciate in the critical care setting, with a sudden drop in hemoglobin being the only obvious manifestation. The purpose of this manuscript is to highlight a rare association between spontaneous retroperitoneal iliopsoas hematoma and myasthenia gravis, and to encourage the prompt recognition and management of these major bleeding events.

## Case presentation

An 86-year-old male with a past medical history significant for type II diabetes mellitus, coronary artery disease (CAD) on 81 milligrams (mg) aspirin, hypertension, and dysphagia status-post esophageal dilation in 2018 presented to the emergency department with a chief complaint of neck weakness and dysphagia for three days. Of note, the patient was also seen one day prior to admission for an acute community-acquired urinary tract infection for which he was prescribed oral cephalexin.

Initially, an intravenous (IV) course of piperacillin-tazobactam was initiated due to concern for aspiration. Dysphagia was believed to be oropharyngeal in etiology, thus neurology was consulted. Workup including acetylcholine receptor binding antibodies (ACHR-Ab), anti-muscle specific kinase (anti-MuSK) antibodies, acetylcholine receptor modulating antibodies, voltage-gated calcium channel antibody, ganglioside monosialic acid (GM1) antibody, and paraneoplastic antibody was initiated due to high clinical suspicion for MG. Electromyography demonstrated early recruitment, decreased amplitudes, and polyphasic potentials in all tested muscles of the left upper extremity consistent with myopathic disorder. The patient was started on empiric pyridostigmine but was intubated two days later due to an inability to protect his airway. A 1-gram three-day course of methylprednisolone was initiated due to suspicion for myasthenic crisis and the patient was started on 40 mg of subcutaneous enoxaparin once daily for deep venous thrombosis (DVT) prophylaxis.

**Figure 1:**
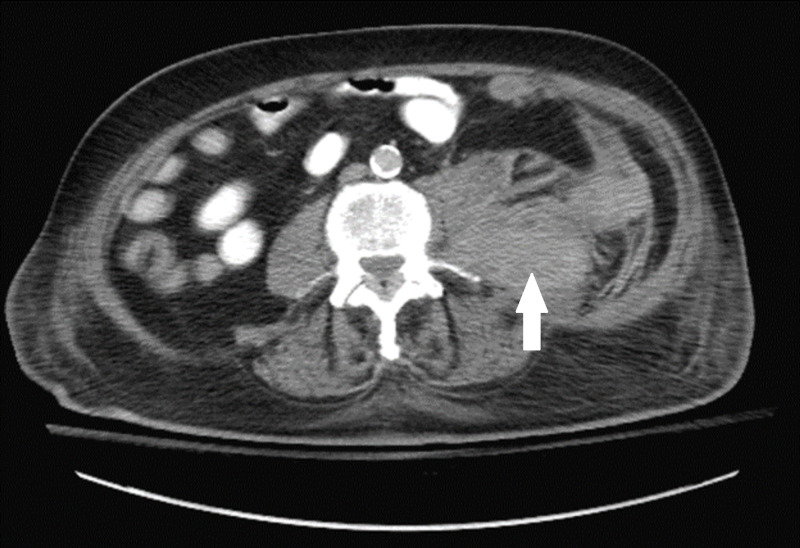
New sizable left retroperitoneal hematoma measuring 10.2 x 6.7 cm extending from the level of the renal mid poles into the iliopsoas region; areas of high density indicate that it is relatively fresh and ongoing hemorrhage cannot be excluded.

The patient was extubated but decompensated into acute hypoxic, hypercapnic respiratory failure secondary to neuromuscular weakness and was reintubated. At this time, the patient experienced an acute drop in hemoglobin (Hgb) from 15.3 g/dL to 12.8 g/dL. A contrast-enhanced computerized tomography (CT) scan of the abdomen and pelvis was performed to rule-out MG due to paraneoplastic syndrome which showed an incidental left retroperitoneal hematoma measuring 10.2 x 6.7 x 12 centimeters (cm) from the renal mid pole to the left iliopsoas with areas of increased density suggestive of active bleeding (Figure 1). There was no known trauma. Blood work was significant for a platelet level of 95 x 103 per mm3, international normalized ratio of 1.1, prothrombin time of 13 seconds, and a partial thromboplastin time of 37.7 seconds. At this time, enoxaparin and aspirin were discontinued to prevent further bleeding. A CT angiogram of the abdomen was performed 24 hours later which demonstrated an interval increase in the size of the hematoma to 15.2 cm in the largest dimension. Hgb values continued to trend downward to a low value of 7.2 g/dL over the next 48 hours. An additional CT scan of the abdomen demonstrated a further interval increase in size of the hematoma to 20 cm in the largest dimension. He was transfused two units of packed red blood cells for which Hgb stabilized; vasopressors were intermittently required to maintain adequate mean arterial pressure. Interventional radiology and vascular surgery teams recommended no surgical intervention.

The patient was subsequently diagnosed with MG with acetylcholine receptor binding antibody of 42.4 nmol/L, acetylcholine receptor modulating antibodies 92%, acetylcholine receptor blocking antibodies 69; anti-MuSK, paraneoplastic, voltage-gated calcium channel, and GM1 antibodies were negative. He was treated with a course of intravenous immunoglobulin and IV corticosteroids without plasmapheresis but experienced a lengthy, complicated stay in the medical intensive care unit, likely complicated by the hematoma.

## Discussion

Spontaneous retroperitoneal and iliopsoas hematomas are associated with a significant mortality increase in the ICU setting, with some studies demonstrating a rate as high as 50% compared to 22% without similar bleeding [1]. Commonly, they are associated with abdominal or pelvic trauma, but are often in the setting of anticoagulation, antiplatelet therapy, iatrogenic causes, known bleeding disorders or vasculitis [2,3]. Frequently, these patients are excessively anticoagulated as demonstrated by routine blood tests, however, some cases occur in the absence of anticoagulation or antiplatelet therapy altogether [4,5]. Recent retrospective studies have identified other significant associations such as advanced age, body mass index >30, and renal dialysis [1].

While some cases of MG have been associated with vascular pathology, to the best of our knowledge no cases of spontaneous iliopsoas hematoma associated with myasthenic crisis have been reported in the literature [6]. MG has an incidence of 0.21 - 2 per 100,000, and while bulbar symptoms including dysphagia, slurred speech, and weakness are the hallmark of the disease, associations with coagulopathy or major bleeding is not well documented [7]. Our patient had no history of trauma. His only anticoagulant was low-dose DVT prophylaxis with enoxaparin and home antiplatelet therapy due to a history of coronary artery disease (CAD). The only obvious clinical sign was acute anemia. A focus on early diagnosis with CT scan and prompt removal of anticoagulation and antiplatelet therapy is crucial, especially in the ICU setting. Transfusion should additionally be considered, and surgical intervention may be warranted. Further studies associating MG with major bleeding events may be beneficial to the management of both conditions.

## Conclusions

Spontaneous retroperitoneal and iliopsoas hematomas are important pathologies to consider when caring for patients in the ICU setting. Often, an acute drop in hemoglobin is the only clinical sign. Prompt recognition and appropriate workup should be pursued. The association between myasthenic crisis and major bleeding events such as spontaneous hematoma are not well documented. Further studies associating these two pathologies may be beneficial to both improve patient outcomes and better manage critically ill patients affected by these conditions.
